# Advanced Calciphylaxis in a Patient With End-Stage Renal Disease: A Case Report Highlighting Diagnostic and Therapeutic Challenges in Late-Stage Presentation

**DOI:** 10.7759/cureus.68866

**Published:** 2024-09-07

**Authors:** Priam P Chaganlal, Varun Kalandoor, Daniel T Jones, Christopher Pace, Scott A Silver

**Affiliations:** 1 Internal Medicine, Touro University Nevada, Henderson, USA; 2 Internal Medicine, Valley Hospital Medical Center, Las Vegas, USA

**Keywords:** chronic kidney disease, esrd, high morbidity, sepsis, skin necrosis, gas gangrene, small vessel disease, vascular calcification, calcific uremic arteriolopathy, calciphylaxis

## Abstract

Calciphylaxis, also known as calcific uremic arteriolopathy, is a rapidly progressive, rare, and severe condition characterized by vascular calcification and skin necrosis. The pathophysiology involves cutaneous arteriolar calcification followed by subsequent tissue ischemia and infarction, which eventually causes extremely painful skin lesions. The condition is associated with substantial morbidity due to severe pain, non-healing wounds, increased susceptibility to infections, and frequent hospitalizations. Calciphylaxis is a highly fatal condition with one-year mortality rates greater than 50%, most frequently due to sepsis.

This report presents a case of a 63-year-old male with end-stage kidney disease (ESKD) who presented with altered mental status and was found to have notable necrotic skin ulcers on the bilateral anterior thighs, a stage IV sacral decubitus ulcer, and necrotic lesions on the scrotum and penis. This case underscores the importance of maintaining a high clinical suspicion for rare conditions like calciphylaxis in patients with multiple risk factors. Diagnosing the disease earlier in its course may improve outcomes and overall prognosis. Unfortunately, in this case, the patient presented too late into the disease course, and ultimately discussions/placement with palliative care were undertaken.

## Introduction

Calciphylaxis is a rare and severe condition characterized by vascular calcification and skin necrosis. The disease mainly affects the medial layer of small arteries and arterioles by causing calcification. This calcification leads to reduced blood flow, endothelial damage, and the formation of microthrombi, which narrow and occlude blood vessels. These vascular changes can result in tissue ischemia, necrosis, and ulceration [1,2]. The progression from vascular calcification to painful, non-healing skin ulcers represents the devastating clinical manifestation of this condition.

Despite its severe nature, the exact causes and mechanisms of calciphylaxis remain poorly understood. Several factors are believed to contribute to its development, including increased parathyroid hormone levels, high calcium and phosphate levels, and the use of activated vitamin D [1,2]. Deficiencies in vascular calcification inhibitors, such as matrix Gla protein and fetuin-A, might also play a role [3]. The condition is thought to be related to abnormalities in bone mineral parameters, particularly in patients with end-stage renal disease (ESRD) on hemodialysis [1,2]. However, calciphylaxis can occur even with normal levels of parathyroid hormone, phosphorus, and calcium, highlighting the complex and poorly understood pathophysiology of the disease [4].

Calciphylaxis is associated with significant morbidity and mortality, particularly in patients with ESRD. The severe nature of the condition, characterized by painful skin ulcers and a high risk of infection, contributes to a mortality rate often ranging between 45% and 80% within the first year of diagnosis [2,4]. The systemic involvement, including possible effects on organs such as the eyes, lungs, and brain, further complicates the clinical picture, making early diagnosis and intervention crucial for improving outcomes [5]. Timely intervention is essential, as delayed diagnosis can lead to rapidly worsening clinical conditions and limited treatment options [2,5].

This case report aims to highlight a particularly severe presentation of calciphylaxis in a patient with multiple comorbidities, including ESRD, recent cardiac surgery, and severe malnutrition. The report seeks to discuss the challenges encountered in managing such a complex case, emphasize the importance of early detection, and explore the implications for clinical practice. This case also serves to underscore the broader clinical relevance of recognizing calciphylaxis early in high-risk patients to improve outcomes.

## Case presentation

The patient is a 63-year-old male with a past medical history, including end-stage renal disease (ESRD) on hemodialysis, coronary artery disease status post-coronary artery bypass grafting (CABG) two months prior, insulin-dependent diabetes mellitus (IDDM), and hypertension. He had been on peritoneal dialysis for the last 30 months. He presented to the hospital with altered mental status, which had progressively worsened over the previous two days. His family reported that while at a skilled nursing facility (SNF), he had become increasingly lethargic and non-verbal, missed several dialysis sessions at his own request, and developed extensive worsening of his chronic wounds.

The patient has a significant surgical history, including a CABG performed six months ago, and a left heart catheterization two days prior to the CABG. Additionally, he underwent an esophagogastroduodenoscopy (EGD) five months ago and had a chest tube placed in his right chest during a previous hospital stay.

The patient receives daily dialysis as part of his treatment regimen for ESRD. The patient's most recent dialysis session was performed using a central venous catheter (CVC) in the chest wall, which had been in place for six days. The dialysis treatment was conducted for a duration of three hours and 30 minutes, utilizing an Optiflux 180NR dialyzer. The blood flow rate (BFR) was maintained at 500 mL/min, with a dialysate flow rate (DFR) of 500 mL/min. The ultrafiltration (UF) rate was set within a range of 1000 to 2000 mL, with a target fluid removal of 1300 mL. The patient’s pre-treatment vitals were stable, with a blood pressure of 114/60 mmHg, a pulse of 85 bpm, and a temperature of 36.4°C.

**Figure 1:**
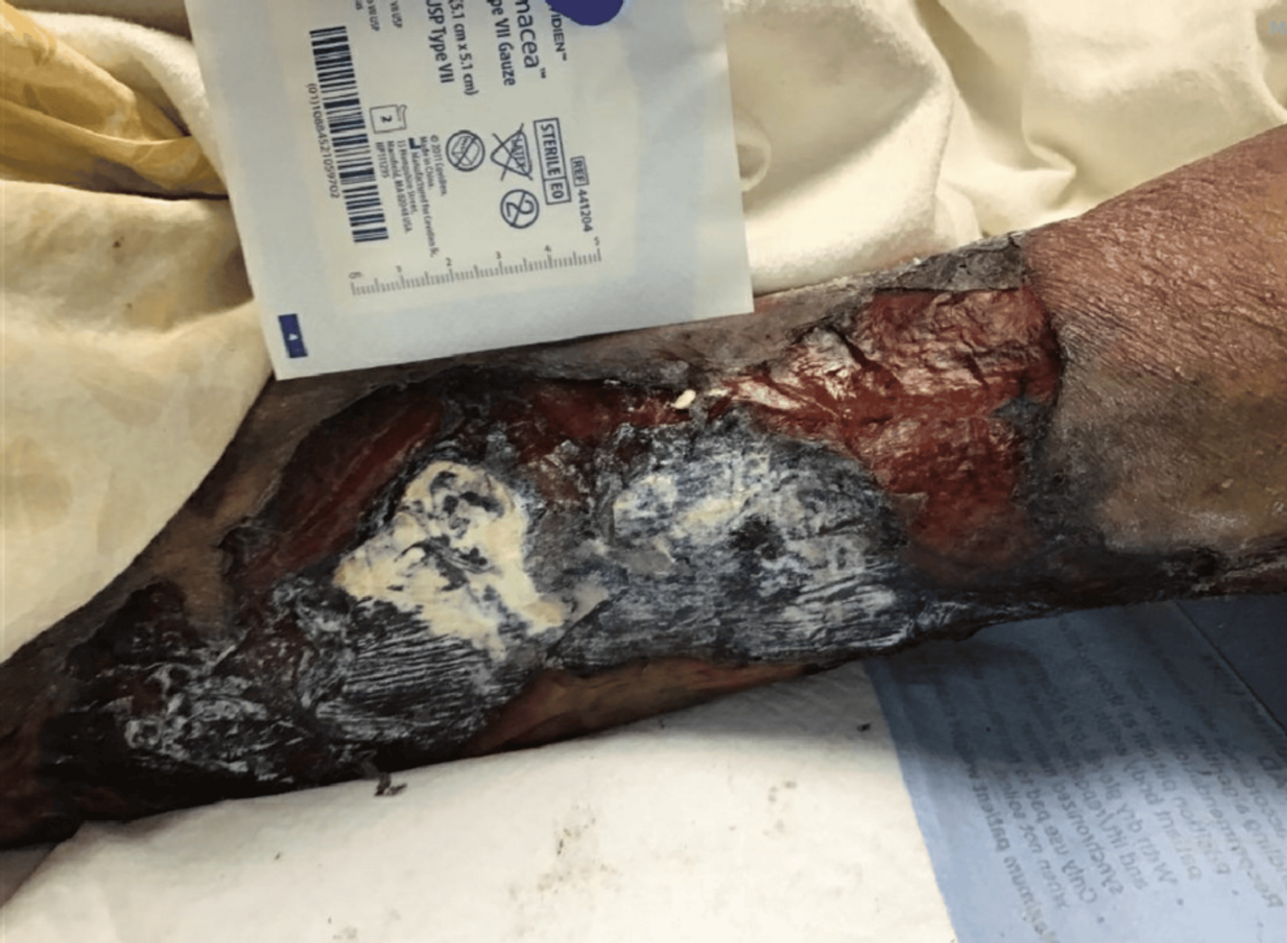
Left anterior thigh showing extensive necrotic skin lesions and ulceration in a patient with advanced calciphylaxis

**Figure 2:**
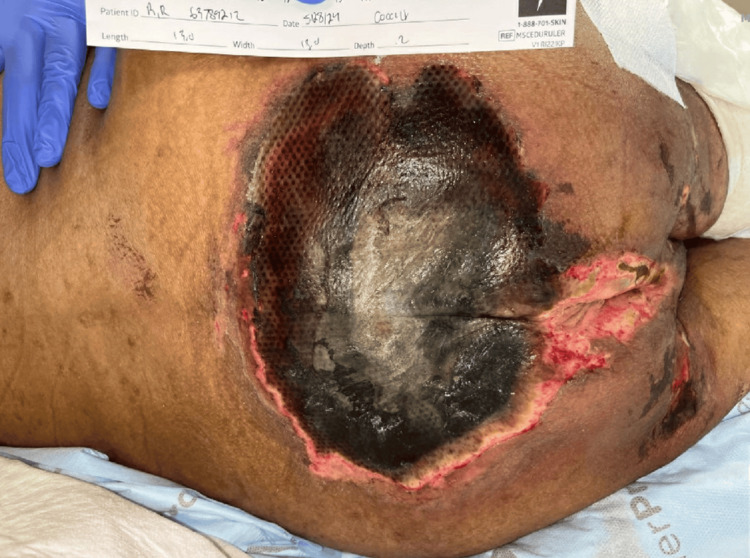
Stage IV sacral decubitus ulcer with purulent discharge in a patient with advanced calciphylaxis

**Figure 3:**
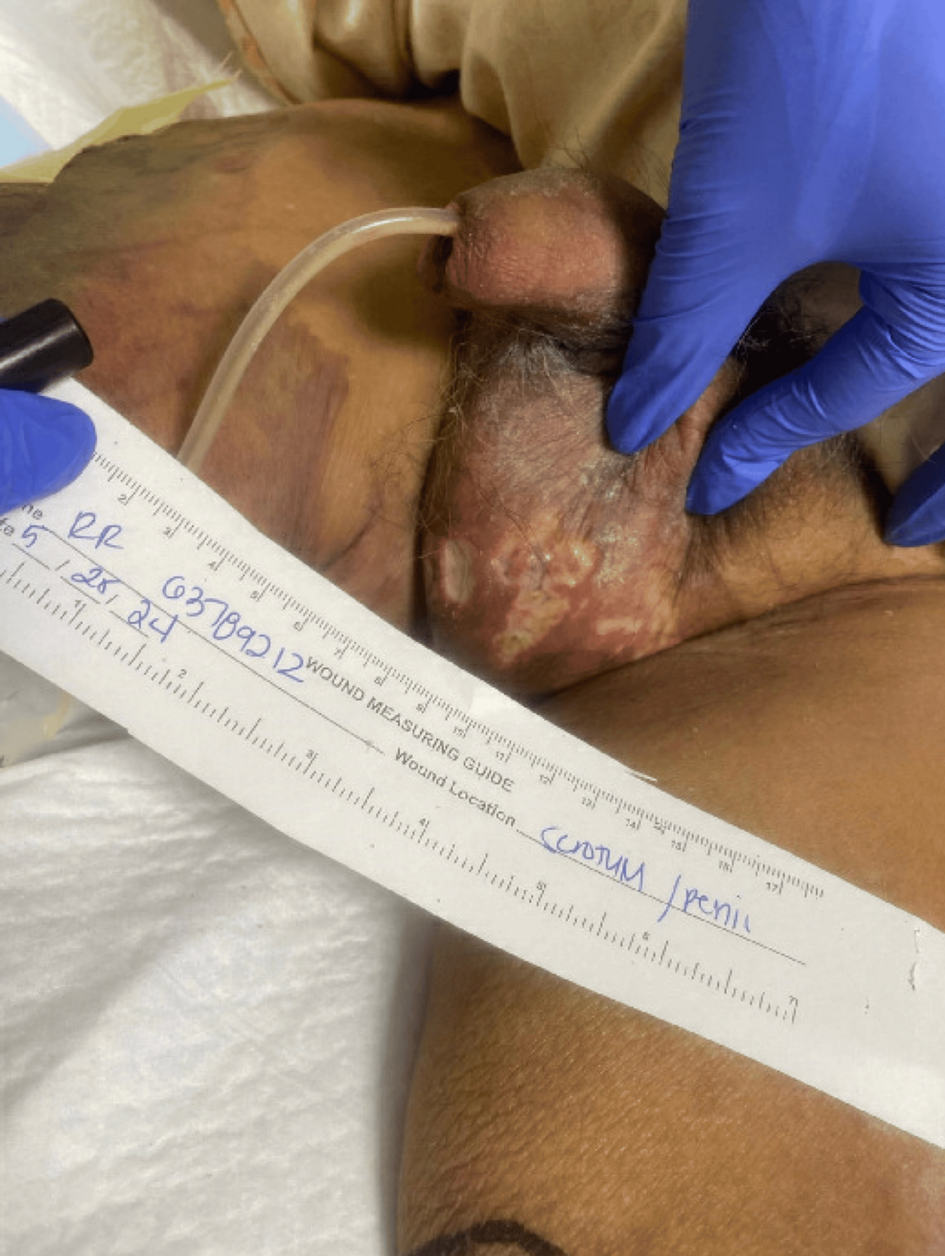
Necrotic lesions on the scrotum and penis, with involvement of the glans in a patient with advanced calciphylaxis

Notably, the patient had significant necrotic skin lesions consistent with calciphylaxis, including large necrotic ulcers on the bilateral anterior thighs, a stage IV sacral decubitus ulcer with purulent discharge, and necrotic lesions on the scrotum and penis, with involvement of the glans (Figures 1, 2, 3).

On physical examination, the patient appeared frail and chronically ill, with a body mass index (BMI) of 22.38 kg/m². He was alert and oriented to person and place but was disoriented to time. There was significant temporal and buccal wasting, indicative of severe malnutrition or cachexia. His cardiovascular examination was notable for a regular heart rate and rhythm, with a large, vertical, recently healed surgical scar from his recent CABG. The patient was not on warfarin. Respiratory examination revealed clear lung fields bilaterally, with normal respiratory effort. Abdominal examination showed mild distention but was otherwise unremarkable. Genitourinary examination revealed a slightly swollen penis with a Foley catheter draining purulent fluid, and the presence of weeping ulcers on the scrotal skin.

**Figure 4:**
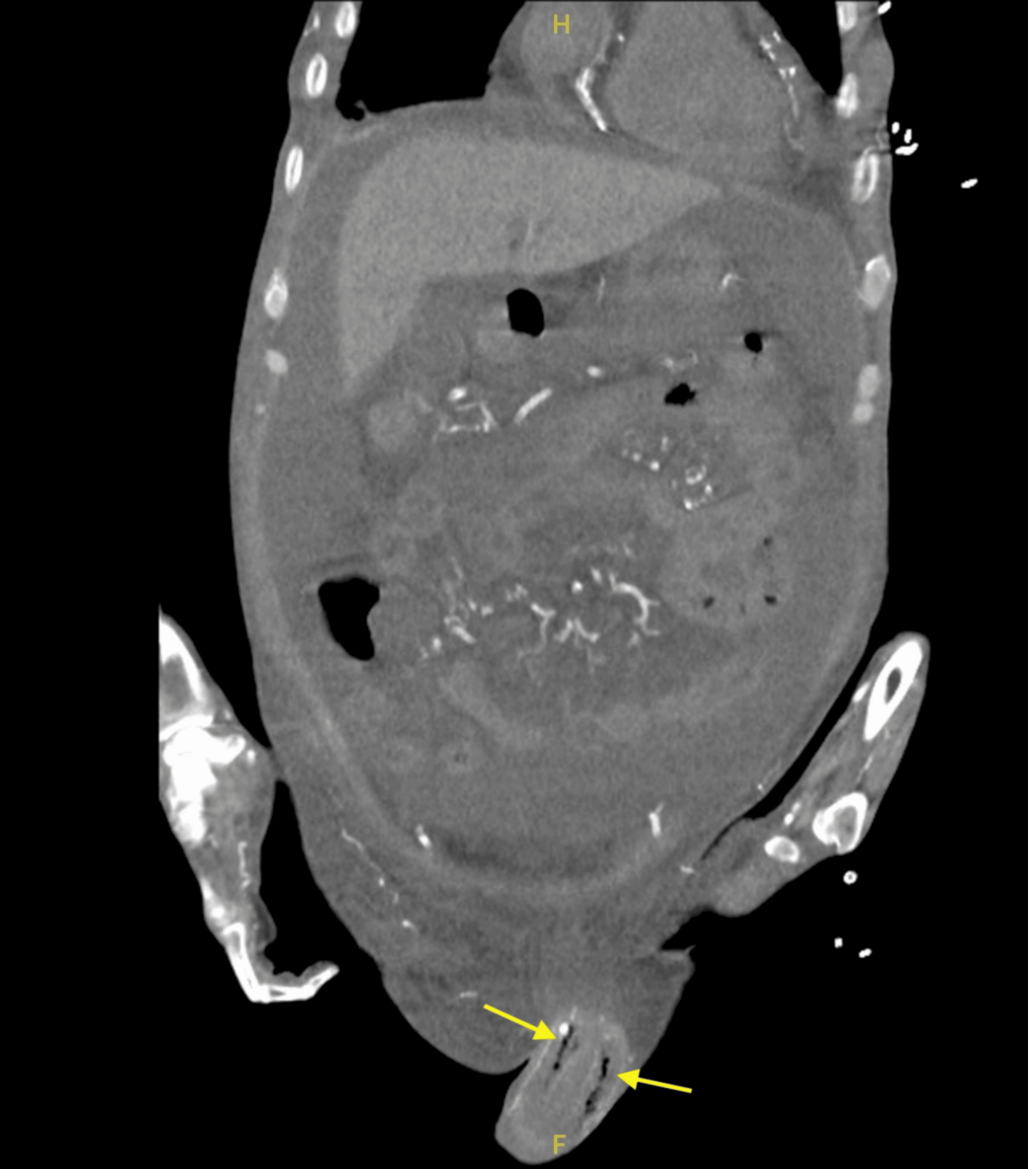
Coronal CT abdomen and pelvis without contrast showing subcutaneous gas within the corpus cavernosum of the penis extending to its base, with diffuse penile edema

**Figure 5:**
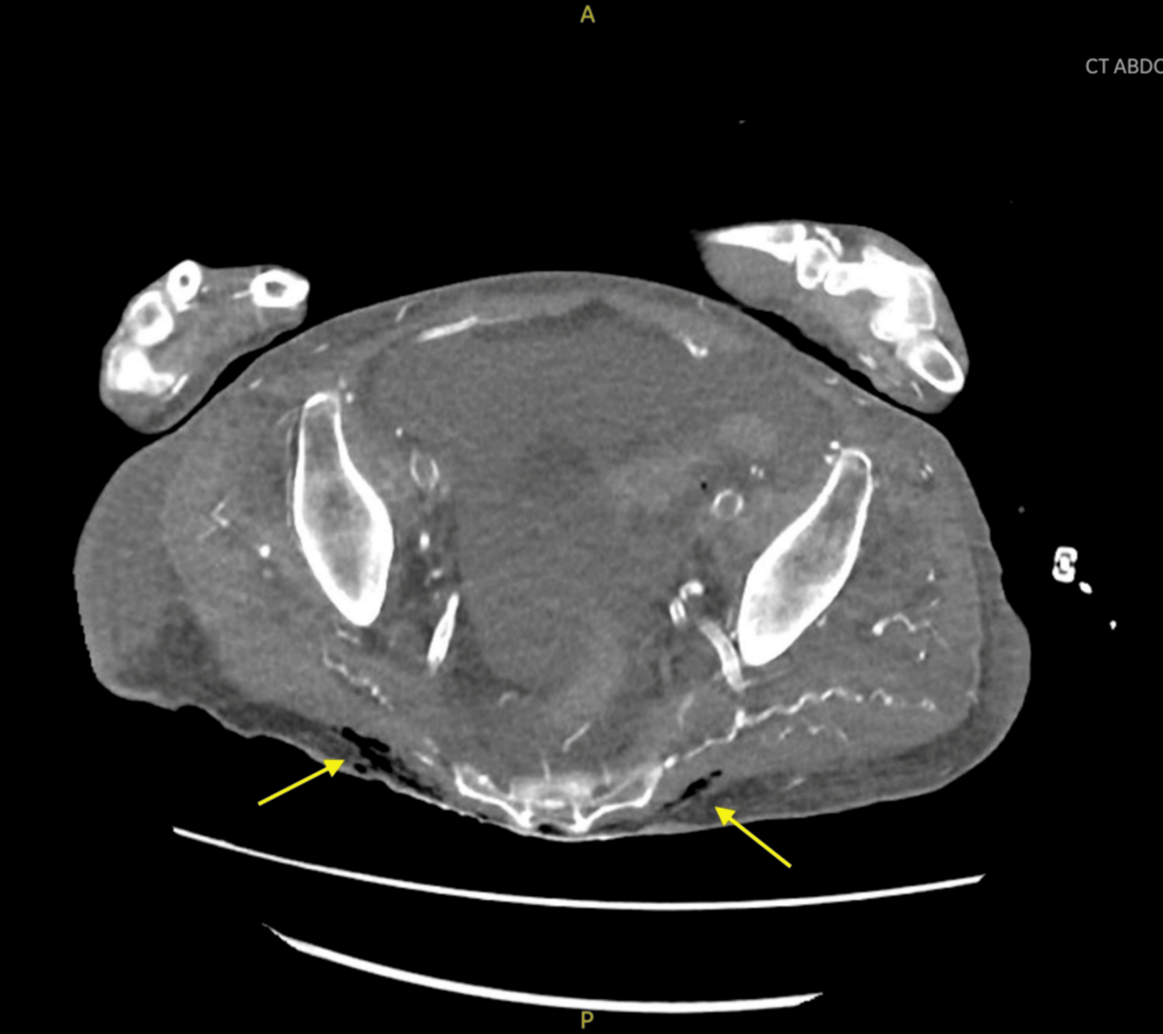
CT abdomen and pelvis without contrast showing subcutaneous gas along fascial planes in sacrum, coccyx, and right hip in a patient with decubitus ulcer

Imaging studies, including a CT scan of the abdomen and pelvis, revealed gas within the soft tissues, including the penis (extending into the corpus cavernosum), sacrum, and coccyx, consistent with severe soft tissue infection or necrotizing fasciitis (Figures 4, 5). 

These imaging findings, particularly the presence of gas within the soft tissues, were alarming and indicated an advanced stage of infection, complicating the overall clinical picture. Laboratory tests were conducted upon the patient's arrival (Table 1).

**Table 1:** Laboratory findings on admission in a 63-year-old male with calciphylaxis and ESRD ESRD, end-stage renal disease

Lab test	Result	Reference range	Notes
Calcium	6.8 mg/dL	8.5-10.2 mg/dL	Low
Phosphorus	2.3 mg/dL	2.5-4.5 mg/dL	Low
Albumin	1.9 gm/dL	3.5-5.0 gm/dL	Low
Total protein	5.2 gm/dL	6.4-8.3 gm/dL	Low
BUN	32 mg/dL	7-20 mg/dL	High
Creatinine	4.26 mg/dL	0.6-1.2 mg/dL	High
Lactic acid	2.6 mmol/L	0.5-2.2 mmol/L	High
Alk Phos	136 units/L	44-147 units/L	High
WBC	22.49x10^3^/mcL	4.0-11.0x10^3^/mcL	High
Hgb	5.4 gm/dL	13.5-17.5 gm/dL	Critically low
Hct	19.4%	41.0-53.0%	Low
BNP	1,305 pg/mL	<100 pg/mL	High
eGFR	15 mL/min/1.73 m^2^	>60 mL/min/1.73 m^2^	Low

Blood cultures subsequently grew Citrobacter freundii complex and Morganella morganii, confirming the presence of polymicrobial bacteremia. Despite the severity of his condition, the patient was hemodynamically stable, without the need for pressor support.

Given the presence of calciphylaxis, a condition marked by vascular calcification leading to ischemia and necrosis of the skin, the orthopedic team determined that the patient’s extensive wounds were unlikely to heal with a hemipelvectomy. His overall clinical picture was further complicated by his ESRD, recent cardiac surgery, and severe malnutrition, which significantly reduced his healing potential. The combination of extensive soft tissue involvement, subcutaneous emphysema, and bacteremia pointed to a poor prognosis, necessitating a careful discussion of goals of care and consideration of palliative options. The patient was transferred to a home hospice.

## Discussion

Calciphylaxis is a rare but severe condition characterized by the deposition of calcium in the small- and medium-sized blood vessels of the skin and subcutaneous tissues [1,2]. This pathological calcification leads to vascular damage, thrombosis, and subsequent ischemia, resulting in painful skin ulcers and necrosis [1,3]. The incidence of calciphylaxis in dialysis patients ranges from 0.4% to 4%, with recent studies indicating an increasing trend, particularly among patients with ESRD on chronic hemodialysis [1,4]. For instance, a nationwide study conducted in the Fresenius Medical Care North America (FMCNA) dialysis units reported a calciphylaxis incidence rate of 3.49 per 1000 patient-years among patients with ESRD on chronic hemodialysis [4].

The pathogenesis of calciphylaxis is complex and multifactorial, involving a combination of factors such as hyperparathyroidism, disturbances in calcium and phosphate metabolism, and endothelial dysfunction [1,3]. Patients with chronic kidney disease, especially those on dialysis, are particularly susceptible due to their compromised ability to regulate these electrolytes and the frequent use of calcium-based phosphate binders [2,4]. Despite these known risk factors, calciphylaxis can still occur in the absence of kidney disease, though most cases are linked to advanced renal failure [5].

Calciphylaxis is associated with several risk factors that significantly contribute to its development and progression. The condition is most commonly seen in patients with ESRD, where compromised renal function disrupts calcium and phosphate metabolism, fostering conditions favorable for vascular calcification [3,4]. Demographically, calciphylaxis predominantly affects Caucasian women, although it can occur across different ethnicities and sexes [4]. Various comorbidities heighten the risk of calciphylaxis, including obesity, diabetes mellitus, hypoalbuminemia, autoimmune conditions, liver disease, and malignancy [5]. Additionally, certain medications, such as warfarin, corticosteroids, calcium-based phosphate binders, activated vitamin D, and iron therapy, are linked to an increased risk of developing calciphylaxis [5]. Furthermore, hypercoagulable states can contribute to the disease’s pathogenesis by promoting vascular thrombosis and ischemia [6].

Clinically, patients with calciphylaxis typically present with extremely painful ischemic cutaneous lesions or subcutaneous nodules, which can occur even before visible skin changes [7]. These lesions commonly manifest as violaceous or erythematous nodules and plaques, which may progress to ulceration, necrosis, and eschar formation [7]. Proximal areas with abundant adipose tissue, such as the abdomen, thighs, and buttocks, are most frequently affected, though distal sites like the digits can also be involved [4]. The extreme pain associated with these lesions is a hallmark of calciphylaxis, significantly impacting patient quality of life [7]. While the skin manifestations are the most prominent, calciphylaxis is a systemic condition that may also affect other organs, including the eyes, penis, muscles, brain, intestines, and lungs, highlighting its severe and widespread impact [3].

Calciphylaxis is diagnosed based on a high index of suspicion, typically confirmed through a skin biopsy of a lesion. Histologic evaluation reveals characteristic findings such as medial calcification of dermal arterioles or small arteries, fibrointimal hyperplasia, microthrombi, and vascular narrowing or occlusion with associated necrosis [8]. These histopathological features are crucial for differentiating calciphylaxis from other skin lesions, particularly in its early stages [8]. However, given the risks associated with a skin biopsy, such as ulceration, bleeding, infection, or necrosis, a comprehensive laboratory workup is crucial, especially when the overall clinical presentation is clear enough to potentially avoid the biopsy [9]. This workup includes a metabolic panel, liver function tests, partial thromboplastin time, international normalized ratio, and albumin levels [9]. Experimental diagnostic tools, such as nuclear bone scans to assess soft tissue calcification and measurements of circulating fetuin-A and matrix Gla protein levels, may also aid in the diagnostic process [10].

The management of calciphylaxis is challenging and involves a multifaceted approach aimed at optimizing renal function, controlling mineral metabolism, and addressing symptoms [7]. Ensuring that dialysis meets adequacy criteria is crucial, with adjustments to dialysis frequency as needed to enhance the clearance of uremic toxins [11]. Careful management of calcium and phosphorus levels is essential; hypercalcemia must be avoided, and phosphorus levels should be maintained below 5.5 mg/dL [11]. Medications that may exacerbate the condition should be discontinued, and calcium supplementation and calcium-based phosphate binders should be replaced with non-calcium-based alternatives [12]. The decision to use anticoagulation with warfarin must be carefully weighed, given the associated risks and benefits [13]. Maintaining parathyroid hormone levels between 150 ng/mL and 300 ng/mL is important, and cinacalcet, a calcimimetic agent, should be used to manage these levels instead of activated vitamin D [14]. Sodium thiosulfate, administered intravenously during dialysis, is believed to aid in treatment through calcium chelation, though its exact mechanism of action remains unclear [14]. Comprehensive care also includes meticulous wound management, pain control, and, if necessary, surgical debridement to address affected areas [15].

Early diagnosis is key to increasing the cure rate and improving prognosis [7]. Attention to atypical skin lesions, leveraging new radiologic technologies, and conducting standardized pathological examinations of skin biopsies in a timely manner are vital strategies for the early diagnosis of calciphylaxis [16]. The "Zhong Da Diagnostic Approach," developed by the Institute of Nephrology of Southeast University, stratifies dialysis patients with clinical suspicion of calciphylaxis into "suspected diagnosis," "clinical diagnosis," and "confirmed diagnosis" [17]. This hierarchical approach to diagnosis emphasizes early detection and has shown promise in improving survival rates and prognosis in patients with calciphylaxis [17].

In one study utilizing the "Zhong Da Diagnostic Approach," 51 Chinese dialysis patients were followed, and an early diagnosis rate of 29.4% (15/51 cases) was achieved. Patients who received early diagnosis and treatment with sodium thiosulfate-based comprehensive therapy demonstrated a higher survival rate, with 87.1% surviving after nine months of follow-up, compared to historical data showing one-year mortality rates of 45-80% [17]. While these findings are encouraging, the study's limited sample size and population suggest a need for further research to validate the generalizability of these results [17].

Without early detection, the prognosis for most patients with calciphylaxis is poor [18]. Lesions are challenging to treat and often refractory to isolated therapy [18]. An interprofessional and multimodal approach is essential, involving a team that includes a nephrologist, dermatologist, dietician, wound surgeon, wound nurse, pain management, and palliative care specialists [18]. This comprehensive approach is necessary to address the complex and multifaceted nature of calciphylaxis [18].

In this particular case, the patient presented with advanced disease, and despite aggressive management, treatment with existing modalities was deemed futile [6]. The patient’s condition was refractory to standard treatments, likely due to the advanced stage of the disease, extensive tissue involvement, and the presence of severe comorbidities [6,7]. This underscores the importance of catching calciphylaxis earlier in the disease process, particularly in patients with multiple risk factors and high clinical suspicion [18]. Ultimately, the decision was made to transition the patient to palliative care, highlighting the need for early intervention to improve outcomes in this challenging condition [18].

## Conclusions

This case emphasizes the value of maintaining high clinical suspicion of calciphylaxis in patients with long-standing ESKD. Although calciphylaxis is a rare condition, it can lead to irreversible complications such as severe skin ischemia, necrosis, and infarction. Early detection is vital as it is a rapidly progressive disease, and later stages of the disease have a very poor prognosis. While skin biopsy remains the mainstay for definitive diagnosis, clinical diagnosis can be made based on a high index of suspicion and the presence of risk factors. Management of calciphylaxis requires the optimization of renal function, which emphasizes the importance of adequate dialysis and optimization of dialysis frequency. Calcium level normalization remains equally important for managing calciphylaxis, as hypercalcemia can exacerbate symptoms and accelerate the disease process. There is evidence to suggest sodium thiosulfate can aid in management due to its calcium-chelating properties, even though its mechanism of action is not clear. Furthermore, an aggressive multidisciplinary approach is necessary as there is no standardized regimen for treatment. Thorough examination and workup of atypical skin lesions in a timely manner remain vital for early diagnosis. This case demonstrated treatment futility in a patient with severe late-stage calciphylaxis with several large necrotic skin ulcers. Future research should focus on creating standardized treatment protocols to improve patient outcomes and increase survivability.
